# Notes and News

**Published:** 1904-09-17

**Authors:** 


### NOTES AND NEWS.

The Duchess of Albany has consented to open the new operating-theatre at the National Hospital for the Paralysed and Epileptic, Queen Square, Bloomsbury, at 3.30 p.m. on Saturday, October 8th, and to attend a harvest festival service in the Chapel at which Bishop Welldon will preach.

Mrs. Barrow has contributed £1,000 to the Liverpool Royal Infirmary for the endowment of a bed in memory of her husband, the late Mr. William James Barrow.

The Sanitary Inspectors' Association held its Autumn Conference at Bournemouth last week. The presidential address was read by Sir James Crichton-Browne, who placed a premium on marriage by stating that married men were less liable to insanity than the unmarried, whilst Dr. Snow announced that married men were longer-lived.

A home in connection with the North-Western Co-operative Convalescent Homes Association has been opened at Otley. The building, which is beautifully situated 700 feet above sea-level on a site of 13 acres, is a mansion adapted for the use of 30 patients. Members of the Association will have special privileges, but the Home will receive other inmates as well.

The Local Government Board are endeavouring to secure a uniform procedure in relation to inspection of tuberculous carcasses of cattle. They think that the recommendations of the Royal Commission on the subject should be followed. This provides that the entire carcass should be seized when tuberculosis is present to a specified degree, and that the affected organs only should be seized when the disease is confined to them in a specified degree.

New regulations have been made at the London Hospital in regard to the out-patients. A small charge is to be made for medicines and dressings, and the names and addresses of the patients are to be open to any local practitioner. Provision is to be made to pay, if necessary, £100 per annum to an almoner provided by the Charity Organisation Society to be stationed in the receiving room to give information as to the standing or means of patients.

A new isolation hospital has been opened at Springhead Meltham for the Colne and Holme districts.

The late Miss Mary Young of South Shields has bequeathed £11,000 to the Ingham Infirmary, part of which is to be devoted to endow a ward to be named after her.

The late Mr. S. A. Duigan, of Kingstown, Dublin, leaves his residuary estate to be divided among the Home for the Dying, St. Michael's Hospital, and the Sisters of the Assumption, all at Kingstown.

The late Mrs. Jessie Palmer, of Regent's Park, has bequeathed one moiety of the ultimate residue of her estate to be expended in endowing beds in large hospitals and convalescent homes to be called the Jessie Alice Palmer Beds.

Last week a small-pox patient escaped from the Dewsbury Isolation Hospital and made his way through the streets to his brother's house. Since the epidemic broke out at Dewsbury in February there have been about 206 cases.

A new isolation hospital has been opened by the Pontypridd District Council at Llantwit Fardre. The hospital is built on terraces, and consists of four separate blocks, two being ward pavilions for 10 patients in each. The cost of the building was £6,700.

Through the will of the late Mrs. Esther Burns of Notting Hill, charitable institutions will benefit to a very large extent. The Samaritan Free Hospital for Women and Children is to receive £5,000; the British Home for Incurables, £3,000, and the Hostel of St. Luke and the Royal Hospital for Incurables £2,000 each.

The David Lewis Epileptic Institution on the colony system is now ready. It is situated at Sandlebridge, near Alderley, and is for the use of sane epileptics. The gift has been made to Manchester by the trustees of the David Lewis Trust. The system combines training and healthy occupation for the inmates of the colony.

The late Mrs. E. Peacock of Salford has bequeathed £1,000 to the Hospital for Sick Children, Pendlebury, to endow a cot in memory of her daughter, Lily Peacock, after whom it is to be named. She also bequeathed £1,000 to the Northern Counties Hospital for Incurables and to the Salford and Pendleton Hospital, £500 to St. Mary's Hospital, Manchester, and £400 to the Cancer Pavillion and Home, Manchester.

A vinegar manufacturer has been fined £5 at Accrington for the sale of a mixture of acetic acid mixed with water under a warranty that the mixture was commercial malt vinegar. There are a good many other "acid" mixtures on the market, which if not unwholesome are certainly not what they claim to be. For instance, we are surprised that those who are now endeavouring to revive the West Indian trade do not go into the question of the composition of many brands of "lime juice" so largely sold. Lime juice is a popular drink in summer. We are under the impression that the West Indian lime growers do not benefit to the extent that they might.

